# Gunshot Wound to the Posterior Fossa With a Transcerebellar Retromesencepahlic Bullet Path, Transient Mutism, and Unexpected Functional Recovery: The Pivotal, Energy-Absorbing Function of the Petrous Bone and Tentorial Leaflet

**DOI:** 10.7759/cureus.37420

**Published:** 2023-04-11

**Authors:** Sahar Sorek, Aaron Miller, Vincy Mathew, Stephanie Moawad, Ralph Rahme

**Affiliations:** 1 College of Osteopathic Medicine, New York Institute of Technology, Old Westbury, USA; 2 Division of Neurosurgery, SBH Health System, New York, USA; 3 Division of Neurological Surgery, SBH Health System, Bronx, USA; 4 Division of Neurosurgery, SBH Health System, Bronx, USA; 5 School of Medicine, City University of New York, New York, USA

**Keywords:** quadrigeminal cistern, tentorium cerebelli, petrous bone, penetrating traumatic brain injury, neuroplasticity, neurotrauma, posterior fossa, cerebellar mutism, gunshot wounds

## Abstract

Given the abundance of vital neurovascular structures, gunshot wounds (GSWs) to the posterior fossa are generally fatal. We present a unique such case where a bullet entered the petrous bone, traversed the cerebellar hemisphere and overlying tentorial leaflet, and reached the dorsal aspect of the midbrain, resulting in transient cerebellar mutism with an unexpectedly favorable functional recovery. A 17-year-old boy sustained a GSW to the left mastoid region with no exit wound and presented with agitation and confusion, ultimately leading to a coma. Head CT revealed a bullet trajectory through the left petrous bone, left cerebellar hemisphere, and left tentorial leaflet, with a retained bullet fragment in the quadrigeminal cistern, overlying the dorsal aspect of the midbrain. Computed tomography venography (CTV) demonstrated thrombosis of the left transverse and sigmoid sinuses and the internal jugular vein. The patient’s hospital course was marked by the development of obstructive hydrocephalus, secondary to delayed cerebellar edema with fourth ventricular effacement and aqueductal compression, possibly worsened by concomitant left sigmoid sinus thrombosis. Following the emergency placement of an external ventricular drain and two weeks of mechanical ventilation, the patient’s level of consciousness improved significantly, with excellent brainstem and cranial nerve function, ultimately leading to successful extubation. Although the patient exhibited cerebellar mutism secondary to his injury, his cognitive abilities and speech improved significantly during rehabilitation. At his three-month outpatient follow-up, he was ambulatory, independent in his daily living activities, and able to verbally communicate using full sentences. Though exceptional, survival and functional recovery may occur after a GSW to the posterior fossa. A basic understanding of ballistics and the importance of biomechanically resilient anatomic barriers, such as the petrous bone and tentorial leaflet, can help predict a good outcome. Lesional cerebellar mutism tends to have a favorable prognosis, especially in young patients with central nervous system plasticity.

## Introduction

Given the high density and abundance of neurovascular structures housed within the posterior cranial fossa, gunshot wounds (GSWs) in that location are generally associated with high rates of mortality, approaching 100% [1]. Moreover, in the unlikely survivors, substantial permanent morbidity often ensues from injury to the brainstem, the vertebrobasilar arterial system, cranial nerves, or the cerebellum [1]. In this report, we describe a unique case of survival with remarkable functional recovery following a GSW to the posterior fossa, including a bullet trajectory through the petrous bone, cerebellar hemisphere, tentorial leaflet, and quadrigeminal cistern, in close proximity to the dorsal surface of the midbrain. We postulate that the biomechanically resilient petrous bone and tentorium acted as natural shock absorbers and anatomic barriers, thereby limiting neurovascular damage. Given that the patient experienced transient cerebellar mutism following this injury, a review of the pathophysiology and neuroanatomic correlates of this rare syndrome is also presented.

## Case presentation

**Figure 1:**
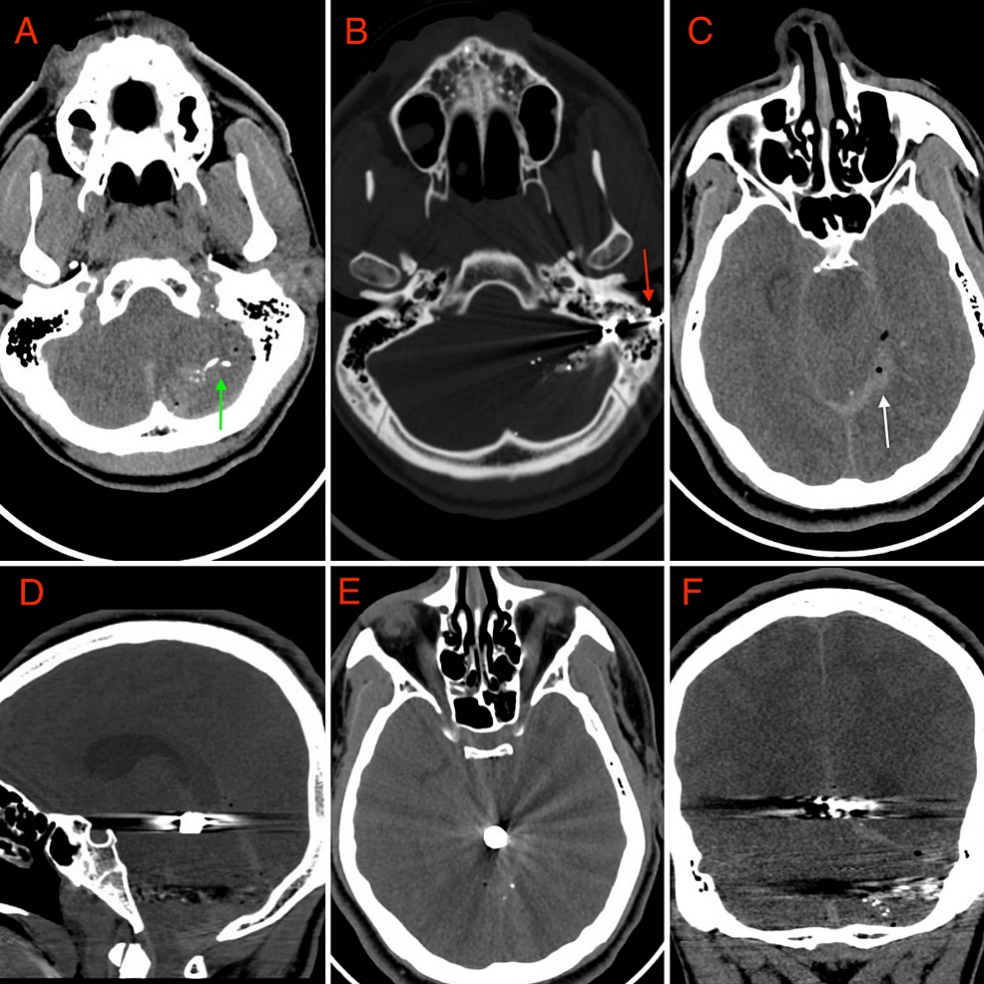
Initial diagnostic imaging following emergency endotracheal intubation (A-F) 1A-B: Axial computed tomography (CT) images showing the bullet’s entrance wound (red arrow) through the left petrous bone with bone fragments and shrapnel in the cerebellum (green arrow); 1C: Axial CT image showing pneumocephalus and hemorrhage (white arrow) along the left tentorial leaflet, demarcating the path of the bullet; 1D-F: sagittal (D), axial (E), and coronal (F) CT images showing bullet rested in the quadrigeminal cistern directly posterior to the midbrain.

A previously healthy 17-year-old boy was brought to the emergency department following a GSW to the head, with a left retro-auricular entry wound in the mastoid region but no obvious exit wound. No history could be obtained regarding the type of firearm used or the distance from where the patient was shot. Though hemodynamically stable, the patient was extremely confused and agitated, however verbal, with a fluctuating level of consciousness, a Glasgow Coma Scale (GCS) score of 11, and pupils being equal, round, and reactive to light. Following emergency endotracheal intubation, a head computed tomography (CT) scan revealed a bullet trajectory through the left petrous bone, left cerebellar hemisphere, and left tentorial leaflet (Figures 1A-1C), with a retained bullet fragment in the quadrigeminal cistern, overlying the dorsal aspect of the midbrain (Figures 1D-1F). Computed tomography venography (CTV) scans demonstrated thrombosis of the left transverse and sigmoid sinuses and the jugular bulb. The patient underwent local debridement of the entry wound and was placed on broad-spectrum intravenous antibiotics, which were continued for 33 days. No surgery was performed in an attempt to remove the bullet.

**Figure 2:**
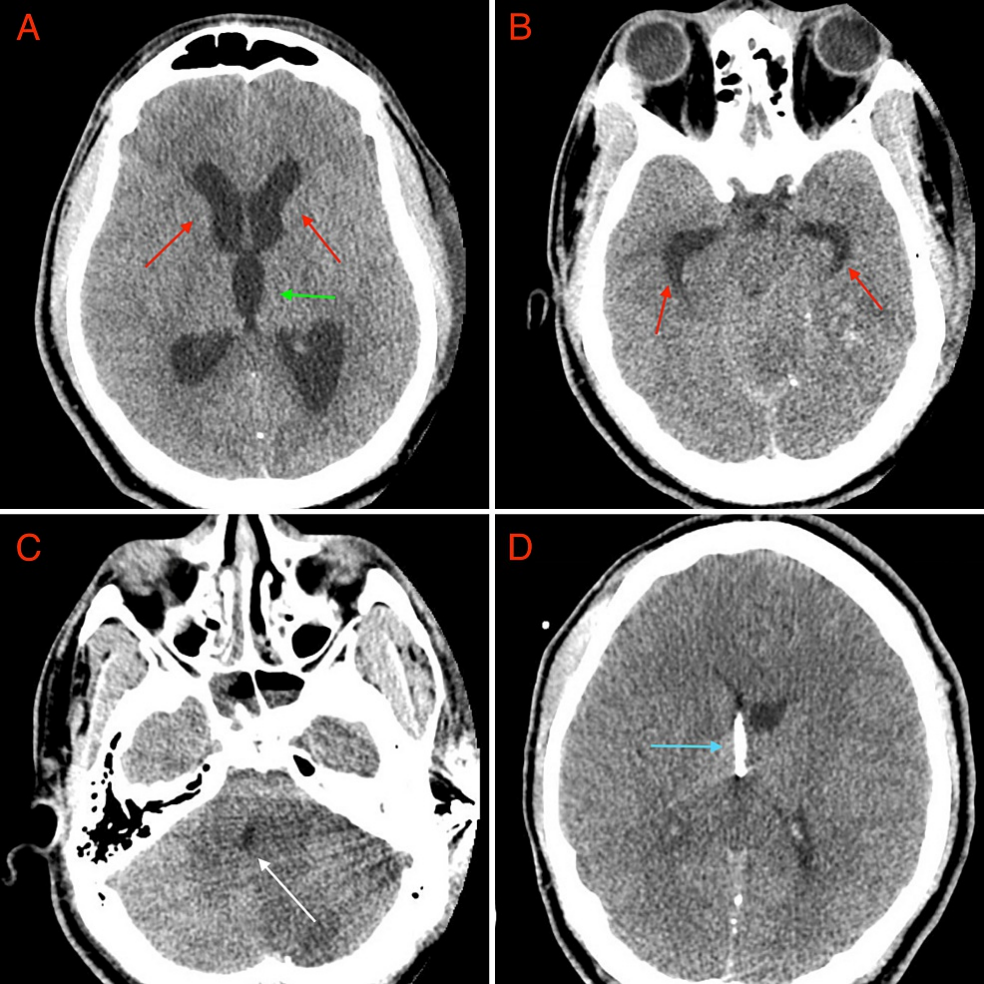
Repeated diagnostic imaging showing cerebellar edema, obstructive hydroecephalus, and external ventricular drain (A-D) 2A-B: Axial CT images showing ventriculomegaly of the lateral (red arrows) and third (green arrow) ventricles secondary to obstructive hydrocephalus; 2C: Axial CT image showing cerebral edema, mass effect, and compression of the fourth ventricle (white arrow); 2D: Axial CT image showing postoperative placement of external ventricular drain (EVD) (blue arrow).

Two days later, the patient exhibited neurologic decline to a GCS score of 7T. Repeat head CT showed worsening left cerebellar hemispheric edema with fourth ventricular and aqueductal effacement, resulting in obstructive hydrocephalus (Figures 2A-2C). Thus, a right external ventricular drain (EVD) was emergently placed to provide temporary cerebrospinal fluid diversion (Figure 2D).

Over the following two weeks, the patient exhibited slow but steady improvement in his level of consciousness, reaching a GCS score of 12T and a nonfocal neurologic exam (spontaneous eye opening, following commands with all four extremities, intact cranial nerves), and was thus successfully extubated. However, following extubation, he was noted to be completely non-verbal in a presentation consistent with cerebellar mutism.

Following a subsequently uneventful hospital course, the patient was ultimately discharged to a rehabilitation facility. He was seen in the clinic three months after injury, where he was noted to have a quasi-normal neurologic exam, except for a slight gait instability and residual left dysmetria, and was remarkably fluent, speaking full sentences.

## Discussion

We present a unique case of survival with a remarkable functional recovery after a GSW to the posterior fossa, in spite of the bullet traveling through the left cerebellar hemisphere and coming in close contact with the dorsal aspect of the midbrain. Moreover, to the best of our knowledge, this is the first-ever reported case of cerebellar mutism resulting from a GSW to the cerebellum.

It is widely reported and agreed upon that GSWs to the head exhibit a high mortality rate. In fact, survival rates after craniocerebral GSWs are in the range of 7-15% overall, with a nearly 90% pre-hospital mortality rate. Among those who make it to the emergency department, mortality rates remain high, at approximately 50% [1]. Although specific outcome data on GSWs to the posterior fossa are severely lacking [2,3], it is generally accepted that given the high density and abundance of important neurovascular structures housed within that small intracranial compartment, severe neurologic compromise and death are often the rule with such injuries [2]. In the largest study to date (N=15), Solmaz et al. report a mortality of 93.3% [3].

In addition to direct injury to neurovascular structures, high-velocity projectiles result in significant shockwave and cavitation effects within the central nervous system, which accounts for the high morbidity and mortality rates seen in this setting [4]. To account for this patient’s unlikely survival and functional recovery, we propose that the unique trajectory assumed by the bullet through a series of biomechanically resilient structures, including the mastoid process, petrous portion of the temporal bone, and thick tentorial leaflet, resulted in gradual deceleration of the projectile and dissipation of its kinetic energy. Thus, by the time it had reached the dorsal aspect of the brainstem, very little energy remained that could be transmitted to the brainstem. In fact, the petrous bone constitutes the thickest and densest bone in the body and has previously been shown to withstand impact forces up to 8,000N [5].

In addition to a remarkable recovery, our patient also exhibited an interesting case of cerebellar mutism. Though cerebellar mutism has most commonly been described in the context of fourth ventricular tumor resections, particularly when an incision is made in the vermis and in children with medulloblastoma [6], this syndrome has only been rarely reported in the setting of trauma (Table 1) and never previously as a result of a GSW to the head [7-12]. The exact pathophysiological mechanism of cerebellar mutism remains unclear, though it is generally agreed upon that damage to the cerebello-cerebral pathways is a major contributing factor [6,13-16]. Moreover, injury to the cerebellar vermis, as in the present case, has been widely suggested as the underlying etiology, especially given that the vermis and adjacent paravermal areas are involved in the coordination of laryngeal functions [17,18]. Interestingly, the characteristic interval of one to five days between the insult of surgery or trauma and the onset of mutism also suggests that direct damage to neural structures may not be the only mechanism involved such as delayed ischemia [13]. In fact, this patient was verbal, though confused, at the time of his initial presentation, prior to endotracheal intubation, and was only noted to have mutism two weeks later, after he was extubated. Finally, it remains unclear whether bilateral cerebellar damage is associated with a higher risk of mutism than unilateral damage [13,19], though, in this case, unilateral damage to the left cerebellar hemisphere and vermis was sufficient to induce transient mutism lasting nearly two months.

**Table 1:** Summary of post-traumatic cerebellar mutism cases reported in the literature CT = computed tomography; F = female; M = male; N/A = not available

Authors (year of publication)	Age (years)	Sex	Type of Injury	Mechanism of Trauma	CT Findings	Time to Onset of Mutism	Clinical Presentation	Time to Resolution of Mutism	Final Outcome
Chivet et al. (2022) [7]	5	F	Blunt	Direct posterior cranial impact from horse's hoof.	Bilateral hemorrhagic contusions of the vermis and roof of the 4th ventricle with intraventricular hemorrhage. Injury to the cerebellar hemispheres and inferior part of dentate nuclei.	2 days (noted after extubation)	Dysarthria, global language repression, irritability, apathy.	12 days	Behavioral symptoms lasted 3 months with resolution.
Chivet et al. (2022) [7]	3	F	Blunt	Fell from a 2.5m high staircase	Contusions to the right cerebellar tonsil, right cerebellum, and right part of the 4th ventricle floor. Injury to the right dentate nucleus.	5 days	Dysarthria and a global regression of language development.	"Few days". Resolved by the 1-month follow-up	Resolution
Lahirish et al. (2021) [8]	8	F	Penetrating	Fallen on a rod that penetrated the neck behind the ear.	Right cerebellum contusion and edema.	3 days	Mute, obeying commands, ataxic gait.	1 month	Regained normal speech.
Kariyatti et al. (2015) [9]	7	M	Blunt	Fell off a cupboard, and the cupboard fell on top of the child.	Comminuted fracture of the right occiput with cerebellar hematoma and fourth ventricle bleeding.	2 days (noted after extubation)	Elective ventilation for 48h. Mutism, gait ataxia, transient upper limb weakness.	1 month	No speech, cranial nerve, motor, or cerebellar deficits.
Fujisawa et al. (2005) [10]	7	M	Blunt	Road traffic accident.	Acute subdural hematoma of the posterior fossa in the left Sylvian cistern, and mass effect of the brainstem.	3 days (noted after extubation)	Mute, following commands.	39 days	Mild ataxia, no cranial nerve palsy.
Koh et al. (1997) [11]	N/A	N/A	Blunt	Motor vehicle accident.	Small contusion of the left cerebellar hemisphere and focal hemorrhage of the left cerebellar peduncle.	N/A	Mutism	N/A	N/A
Yokota et al. (1990) [12]	6	M	Blunt	Struck temporooccipital region during a motor vehicle accident.	Contusion of the left temporal lobe and left cerebellar hemisphere.	N/A	Mutism	N/A	N/A

## Conclusions

In this case, we report the first documented occurrence of cerebellar mutism due to a gunshot wound (GSW) to the head, with surprisingly excellent neurological and functional outcomes. Though this case is exceptional, a favorable prognosis and functional recovery may occur after a high-velocity penetrating trauma to the posterior fossa. We propose that this is due to the biomechanically resilient anatomic structures in the bullet's path, including the thick, petrous portion of the temporal bone and the leaflet of the tentorial dura. In traveling through these structures, the high kinetic energy of the bullet projectile dissipated during its entry and travel within the skull. As a result of this, the cavitation effect and damage typically imposed by GSWs, especially to an area of the skull dense with important neurovascular structures, remained relatively low.
